# Attribution Assessment and Prediction of Runoff Change in the Han River Basin, China

**DOI:** 10.3390/ijerph19042393

**Published:** 2022-02-18

**Authors:** Mengru Wei, Zhe Yuan, Jijun Xu, Mengqi Shi, Xin Wen

**Affiliations:** 1 College of Water Conservancy and Hydropower Engineering, Hohai University, Nanjing 210098, China; 201602010109@hhu.edu.cn (M.W.); njwenxin@163.com (X.W.); 2 Changjiang River Scientific Research Institute, Changjiang Water Resources Commission of the Ministry of Water Resources of China, Wuhan 430010, China; xujijune@mail.crsri.cn; 3 College of Geomatic, Xi’an University of Science and Technology, Xi’an 710054, China; 20210061026@stu.xust.edu.cn

**Keywords:** Han River Basin, Budyko framework, runoff change, land use, cover change

## Abstract

The ecological environment and water resources of the Han River Basin (HRB) are incredibly susceptible to global warming. Naturally, the analysis of future runoff in HRB is believed to offer a theoretical basis for water resources management and ecological protection in HRB. The purpose of this study is to investigate and forecast the effects of climate change and land use change on runoff in the HRB. This study uses CMIP6 data to simulate three future climate change scenarios (SSP126, SSP245 and SSP585) for changes in precipitation and temperature, a CA-Markov model to simulate future land use change scenarios, and the Budyko framework to predict future runoff changes. The results show that: (1) Between 1974 and 2014, annual runoff (*R*) and annual precipitation (*P*) in the HRB decline not so significantly with a rate of 1.3673 mm/a and 1.2709 mm/a, while maximum temperature (*Tmax*) and minimum temperature (*Tmin*) and potential evapotranspiration (*E*_0_) show a non-significantly increasing trend with 0.0296 °C/a, 0.0204 °C/a and 1.3313 mm/a, respectively. Precipitation is considered as main contributor to the decline in Han River runoff, accounting for 54.1%. (2) In the HRB, overall precipitation and temperature are estimated to rise in the coming years, with all other hydrological variables. The comparison of precipitation rise under each scenario is as follows: SSP126 scenario > SSP585 scenario > SSP245 scenario. The comparison of the temperature increase under each scenario is as follows: SSP585 scenario > SSP245 scenario > SSP126 scenario. (3) In the HRB, farmland and grassland land will continue to decline in the future. The amount of forest acreage is projected to decline but not so significantly. (4) The future runoff of the HRB shows an increasing trend, and the future runoff varies in different scenarios and periods. Under the land use scenarios of maintaining LUCC1992–2014 and LUCC2040 and LUCC2060, the *R* change rates in 2015–2040 are 8.27–25.47% and −8.04–19.35%, respectively, and the *R* in 2040–2060 are 2.09–13.66% and 19.35–31.52%. At the same time, it is very likely to overestimate the future runoff of the HRB without considering the changes in the land use data of the underlying surface in the future.

## 1. Introduction

In recent years, global warming has swept through most parts of the world [1]. In some regions, climate change and human activities have induced substantial changes in land use/cover (LUCC), precipitation, and temperature, resulting in significant changes in watershed runoff over the years [2,3,4,5]. These changes may probably cause a wide range of natural, environmental, and economical destruction [6]. The spatial and temporal variability in runoff is an essential component of the hydrological cycle [7,8]. Therefore, it is crucial for regional water resources management and planning to analyze the response of watershed hydrology to LUCC changes caused by climate change and human activities, and to assess the impacts of climate change and land use on runoff and water cycle changes [9,10,11]. In the context of a changing climatic environment, watershed ecohydrology research and watershed soil and water resources management are facing new challenges [12]. The challenge of predicting and responding to the effects of future climate change and human activities on water resources quantity and ecology in Han River Basin (HRB) of China is particularly prominent [13].

Climate change and LUCC change have essential impacts on precipitation and runoff processes [14,15]. In recent years, a great deal of research has been carried out in China on runoff prediction and attribution analysis in changeable environments [16,17]. There are many quantitative calculations about the contribution of runoff change, such as using the Budyko framework to calculate the elastic coefficient of each driving factor and quantitatively evaluate the contribution rate of climate change and human activities to runoff change [18]. In these studies, precipitation and potential evapotranspiration are the dominant factors influencing runoff, regardless of other factors affected by many watershed characteristics [19,20], such as vegetation and anthropogenic impacts, which have significant regional differences in their effects on runoff variability [21,22]. The current widely used method for predicting the runoff response to climate change is the hydrological model method [23,24,25], which mainly uses the global climate models (GCMs) model data input into the hydrological model for hydrological simulation [26,27]. Hydrological models can be essentially divided into Newtonian models and Darwinian models. The Darwinian model treats the hydrological system as a whole by identifying spatial and temporal correlations [28]. The Budyko hydrothermal coupling model is one of the Darwinian models. Usually, Budyko assumes that there is a coupled equilibrium relationship between water and energy in the watershed (called the hydrothermal equilibrium relationship) [29]. Therefore, the future runoff can be predicted through the Budyko water balance equation. In addition, its calculation is simple, the data input is small, and its physical meaning is clear. It is equivalent to other hydrological models under certain conditions. It has been widely used to analyze the impact of climate change on runoff for a long time and has been verified in many river basins. It has been widely used for the impact of climate change on runoff [30,31]. Therefore, the future runoff can be predicted through the Budyko water balance equation.

The Han River, China, is the source area of the South-North Water Diversion Project [32], and future changes in water resources in the HRB will directly affect the efficiency of the development and utilization of the South-North Water Diversion Project [33]. The Han River’s middle and lower reaches, on the other hand, are hearty grain and cotton production centers in China [34]. Affected by global warming, the trend in annual extreme precipitation at the Han River stations has shown variable performance over the last 50 years [35], but the average annual runoff in the HRB shows a declining trend [36]. Form some experts’ perspective, changes in land use in the HRB have a higher impact on runoff throughout the year than during flood season [37]. There have been some previous studies for runoff prediction in the HRB, however, the results are inconsistent due to discrepancies in study indicators, global climate model selection, and other factors. Changes in runoff are actually the result of multiple factors [38]. In previous studies on Budyko, an empirical link between the parameter *n* and vegetation attributes was created using the Budyko framework [18]. The Budyko parameter *n*, to a great extent, is influenced by many environmental factors besides vegetation traits (e.g., soil, geology, topography, etc.) [39]. Given these factors, we established an empirical relationship between the Budyko parameter and subsurface land use change, based on which future hydrological changes were predicted.

Therefore, the overall objectives of this study are: (1) analysis of historical hydrological variables in the HRB from 1974–2014, attribution analysis of runoff changes in the HRB using the Budyko framework, and exploration of the causes of runoff changes; (2) a future scenario of the HRB was constructed to establish a semi-empirical relationship between the Budyko parameter *n* and LUCC, and the Cellular Automata-Markov (CA-Markov) model was used to simulate the land use data in 2040 and 2060 under the current conditions and to calculate the parameter *n* under the future land use scenario; (3) three shared socioeconomic pathway (SSP) scenarios (SSP126, SSP245, and SSP585) based on global climate models and the Budyko water balance method were used to predict future runoff. The overview of this paper is as follows: The materials and methods section introduces the Budyko theory, the bias correction method, and the CA-Markov model, and the results section presents the analysis of historical hydro-meteorological elements and attribution analysis, climate and land use change scenario setting, and future runoff prediction of the HRB. Then, the discussion and conclusion are presented in Section 4 and Section 5.

## 2. Materials and Methods

### 2.1. Study Area

**Figure 1:**
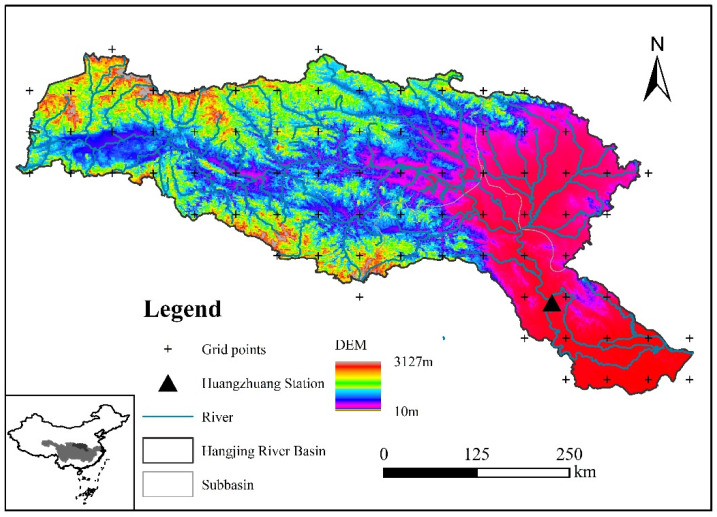
The geographical position of the HRB and meteorological grid points.

The basin is located within 106°15′~114°3′ E and 30°10′~34°20′ N with an area of about 159,000 km^2^. The Han River, as the largest tributary of the Yangtze River, has a total length of 1577 km [26]. The basin’s average yearly temperature is 12–16 °C, average yearly precipitation ranges from 600 to 1100 mm, decreasing from southeast to southwest to northwest, while average annual runoff depth in HRB ranges from 100 to 600 mm. The Han River has a subtropical monsoon climate and abundant water resources. The HRB’s geography is high in the west and low in the east, high in the north and low in the south. The Qinling Mountains to the north and the Daba Mountains to the south define the rugged upper reaches; the Fuyu Mountains to the north, the Wudang Mountains to the south, and the Nanyang Basin to the center lead to the flat middle reaches [40]. The lower reaches of the Han River are the Jianghan Plain with its flat terrain. The basin’s runoff is irregularly distributed throughout the year, with the majority concentrated from May and October, and there will be substantial inter-annual volatility, making it vulnerable to droughts and floods. Figure 1 depicts a schematic representation of the river system and a digital elevation model (DEM) of the HRB.

### 2.2. Variables and Data Sources

(1) Huangzhuang hydrological station is the primary control station for the lower sections of the Han River, its geographical location is shown in Figure 1. The Huangzhuang station runoff data utilized in this study were sourced from the Yangtze River Water Resources Commission’s Hydrological Bureau (http://www.cjh.com.cn/, accessed on 2 March 2021).

(2) Precipitation and temperature data were obtained from the China Meteorological Science Data Sharing Service (CMSDSS). The 0.5° × 0.5° grid point dataset of daily surface precipitation values in China and the 0.5° × 0.5° grid point dataset of daily surface temperature values in China (http://data.cma.cn/, accessed on 4 January 2021) (1961–2020) were used in this study.

(3) LUCC data were obtained from the Environmental Science Data Centre of the Chinese Academy of Sciences land use remote sensing monitoring data with a resolution of 1 km in years 1980, 1990, 1995, 2000, 2005, 2010, and 2015 (http://www.resdc.cn/, accessed on 5 March 2021). Combined with the actual situation of the study area from 1980–2015, farmland, forestland, grassland, water, built, and unused land were identified as the six land uses in the research region. The DEM data were obtained at a resolution of 1 km from the Chinese Academy of Sciences’ Environmental Science Data Centre (http://www.resdc.cn/, accessed on 5 March 2021), and the slope data were generated by processing the DEM with the ArcGIS10.8 toolbox Slope. Referring to the study of Yuan et al. [41], the relevant data were preprocessed in ArcGIS 10.8 software, converted to the same projection and unified at a resolution of 1 km.

(4) The grid data of the mean daily precipitation outputted from 5 global climate models (CanESM5, MRI-ESM2-0, IPSL-CM6A-LR, NESM3, KACE-1-0-G) of CMIP6(Coupled Model Intercomparison Project Phase (6) were used in this paper (https://esgf-node.llnl.gov/search/cmip6/, accessed on 30 May 2021). We have selected these five models to be able to simulate precipitation performance well [42], and provide complete daily climate data (including precipitation, maximum temperature, minimum temperature, etc.) for the future from 2015–2060. Climate models is shown in Table 1. Among the multiple scenarios provided by CMIP6, this study selected the historical (1961–2011) and three shared socioeconomic pathway scenarios (2015–2060): SSP126, SSP245, and SSP585, representing low, medium, and high emission forcing scenarios, respectively. Due to the low spatial resolution of the five selected climate models and the differences between the models, the spatial resolution of all models was standardized to 0.5° × 0.5° using inverse distance weight interpolation, and the interpolated model data were corrected for bias on each grid, the time scale chosen for bias correction in this study is 1961–2011.

For ease of reading, the following hydrologic variables are selected in this paper, as you see in Table 2. At the same time, according to the research content, it is divided into two periods, and the specific division is as follows. This study takes 1974–2014 as the historical period to evaluate the historical hydrological variables. The historical period is divided into two sub-periods, 1974–1991 as the base period, and 1992–2014 as the change period, see Section 3.1.1. This study takes 2015–2060 as the future period to evaluate the future hydrological variables. The future period is divided into two sub-periods: the near-term (2015–2040) and the long-term (2040–2060). The historical period of climate model bias correction is 1961–2011, and the future period is 2015–2060.

### 2.3. Methods

**Figure 2:**
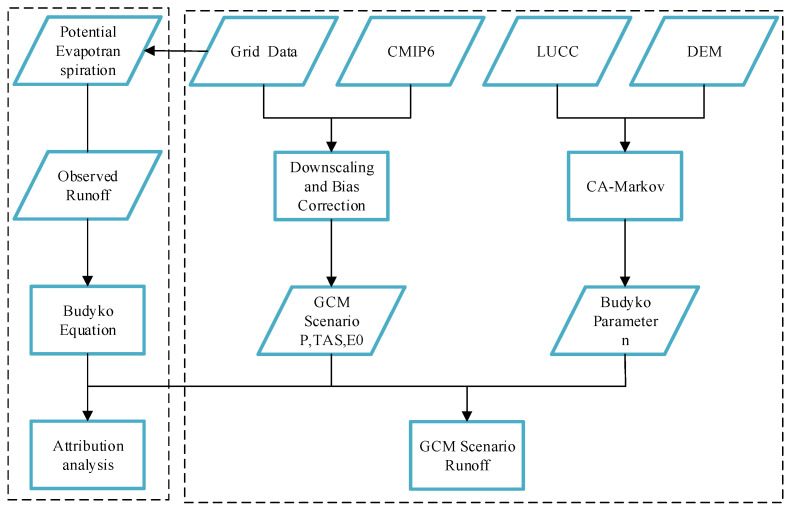
Evolution and prediction of runoff in the HRB based on Budyko hypothesis.

The critical steps in the Budyko-based runoff evolution and prediction in the HRB are as follows: (1) collection of hydro-meteorological data, DEM data, LUCC data, and CMIP6 data. (2) Future climate change scenarios prediction by using statistical downscaling and multi-model ensembles to forecast future climate change sequences. (3) LUCC change prediction by using a CA-Markov model to determine the Budyko parameter *n* in connection to land use under future change scenarios. (4) Prediction of annual runoff in the HRB from 2015 to 2060 under several future change scenarios based on Budyko framework. The specific procedure is shown in Figure 2.

#### 2.3.1. Quantitative Identification of Runoff Changes Based on Budyko’s Hypothesis

(1) Budyko hypotheses-based water balance method.

Budyko’s theory, based on the hydrothermal equilibrium equation, is commonly utilized in ample watershed water and energy balance investigations [43]. Many scholars have introduced subsurface parameters to characterize the influence on the state of coupled hydrothermal equilibrium in watersheds [39]. The Choudhury–Yang equation is used in this paper and its application is relatively broad [8,44], of which Choudhury–Yang is the hydro-energy equation that contains watershed characteristics (including vegetation changes) and their differences in the equilibrium analytic equation [45]. It can be expressed in the following formula:

(1) $$E=\frac{PE_{0}}{{\left(P^{n}+{E_{0}}^{n}\right)}^{1/n}}$$

where *E* is the average annual actual evapotranspiration, *n* is the subsurface parameter related to the land use type; *P* is the precipitation, and *E*_0_ is the potential evapotranspiration, which can be calculated according to the Hargreaves formula recommended by FAO56 [46].

Combining the water balance equation:

(2) R=P−E

A water balance equation such as the following formula can be used to compute the average annual runoff of the basin *R*:

(3) $$R=P-\frac{PE_{0}}{{\left(P^{n}+{E_{0}}^{n}\right)}^{1/n}}$$

where the parameter *n* can be obtained from the *R*, *P*, and *E*_0_ for a given period by Equation (3).

(2) Runoff elasticity based on the Budyko’s hypothesis.

Runoff elasticity, defined by Schaake et al. [47] as the degree of change in runoff per unit change in climate factors, was originally presented in 1990. The precipitation elasticity of runoff is expressed as $\varepsilon_{P}=\frac{dR/R}{dP/P}$, and similarly, we define the potential evapotranspiration elasticity of runoff as $\varepsilon_{E_{0}}=\frac{dR/R}{dE_{0}/E_{0}}$, and the landscape elasticity of runoff as $\varepsilon_{n}=\frac{dR/R}{dn/n}$. The elasticity coefficients for each variable are calculated as follows:

(4) $$\varepsilon_{P}=\frac{1-{\left[\frac{{\left(E_{0}/P\right)}^{n}}{1+{\left(E_{0}/P\right)}^{n}}\right]}^{1/n+1}}{1-{\left[\frac{{\left(E_{0}/P\right)}^{n}}{1+{\left(E_{0}/P\right)}^{n}}\right]}^{1/n}}$$

(5) $$\varepsilon_{E_{0}}=\frac{1}{1+{\left(E_{0}/P\right)}^{n}}\frac{1}{1-{\left[\frac{1+{\left(E_{0}/P\right)}^{n}}{{\left(E_{0}/P\right)}^{n}}\right]}^{1/n}}$$

(6) $$\begin{array}{c} \varepsilon_{n}=\frac{A-B}{{\left[1+{\left(P/E_{0}\right)}^{n}\right]}^{1/n-1}}\\ A=\frac{P^{n}\ln(P)+E_{0}{}^{n}\ln E_{0}}{P^{n}+E_{0}{}^{n}}\\ B=\frac{\ln(P^{n}+E_{0}{}^{n})}{n} \end{array}$$

(3) Runoff attribution based on the Budyko’s hypothesis.

In this study, the annual runoff series at Huangzhuang Station (Figure 1) in the HRB was analyzed using the non-parametric Mann–Kendall test and the Pettitt mutation test. The study cycle is divided into two sub-periods based on the mutation points. Changes in runoff computed as a result of changes in precipitation, potential evapotranspiration, and LUCC are stated as:

(7) $$\Delta R_{P}=\varepsilon_{P}\frac{R}{P}\Delta P$$

(8) $$\Delta R_{E_{0}}=\varepsilon_{E_{0}}\frac{R}{E_{0}}\Delta E_{0}$$

(9) $$\Delta R_{l}=\varepsilon_{n}\frac{R}{n}\Delta n$$

where: $\Delta P=P_{2}-P_{1}$, $\Delta E_{0}=E_{0}{}_{,1}-E_{0}{}_{,2}$, $\Delta n=n_{2}-n_{1}.$ The relative contribution of each factor to runoff is calculated as follows.

(10) $$\begin{array}{l} \eta_{P}=\Delta R_{P}/\Delta R\times 100\%\\ \eta_{E_{0}}=\Delta R_{E_{0}}/\Delta R\times 100\%\\ \eta_{l}=\Delta R_{l}/\Delta R\times 100\% \end{array}$$

where $\eta_{P}$, $\eta_{E_{0}}$ and $\eta_{l\;}$ represent the contributions of precipitation, potential evapotranspiration, and landscape change, respectively.

#### 2.3.2. Climate Change Future Scenario Setting

(1) Bias correction.

GCMs output data are often prone to high systematic biases and may not be directly used in basin-scale hydrological simulations [48]. Hence, after inverse distance weight interpolation, the CMIP6 daily data were corrected using two bias correction methods: the local intensity scaling (LOCI) method and the quantile mapping (QM) method. Of which the LOCI can successfully correct precipitation data: the precipitation day frequency and precipitation intensity. The threshold of simulated precipitation occurrence is set at 0.1 mm in this paper to determine that the threshold of simulated precipitation occurrence, so that the frequency of simulated precipitation occurrence in the historical base period is consistent with the measured series. The threshold is used in future periods to correct the frequency of precipitation occurrence in future periods. The QM is a frequency distribution-based method that considers observed and simulated precipitations to be consistent in frequency distribution [49] and uses empirical cumulative distribution functions (ecdfs) to correct precipitation and temperature in future periods, as well as the frequency of precipitation and temperature occurrences [50]. In this paper, two methods, LOCI and QM, were used to correct the frequency and magnitude of occurrence of daily precipitation series in turn, and the QM was used to correct the temperature.

(2) Taylor diagram.

In order to comprehensively evaluate the simulation effects of climate models before and after bias correction, this paper selects the Taylor diagram to evaluate the simulation effects of the five models’ annual average precipitation, maximum temperature and minimum temperature. The Taylor diagram can intuitively judge the simulation ability of the five models to the measured precipitation, maximum temperature and minimum temperature. Taylor diagrams are essentially an ingenious combination of the model’s correlation coefficient (R), centralized root-mean-square error (RMSE), and standard deviation σ (SD) onto a polar graph. The cosine relationship between the three indicators is based on [51]:

(11) $$R=\frac{\frac{1}{N}\sum_{n=1}^{N}\left(f_{n}-\bar{f}\right)\left(r_{n}-\bar{r}\right)}{\sigma_{f}\sigma_{r}}$$

(12) $$\mathrm{RMSE}={\left[\frac{1}{N}{\sum_{n=1}^{N}\left[\left(f_{n}-\bar{f}\right)-\left(r_{n}-\bar{r}\right)\right]}^{2}\right]}^{1/2}$$

(13) $$\sigma_{f}=\frac{1}{N}\sum_{n=1}^{N}{\left(f_{n}-\bar{f}\right)}^{2}$$

(14) $$\sigma_{r}=\frac{1}{N}\sum_{n=1}^{N}{\left(r_{n}-\bar{r}\right)}^{2}$$

where *f*, *r* represent the measured and simulated values, respectively, $\bar{f}$ and $\bar{r}$ represent the measured and simulated mean values, respectively. $\sigma_{f}$ and $\sigma_{r}$ represent the measured and simulated standard deviation, respectively.

#### 2.3.3. Future Land Use Scenario Setting Based on the CA-Markov Model

The ideas behind Markov forecasting come from the work of the mathematician Markov on stochastic processes. The Markov prediction principle is now widely used in studies of the evolution of land patterns. In the study of land cover evolution, a given period’s land use category can correspond to the possible conditions in a Markov process that is only related to the previous period’s land use category [52,53].

The steps in this article that use CA-Markov are as follows:

(1) Firstly, the measured LUCC 2005 and LUCC 2010 of the HRB were cropped respectively, and the transfer probability matrix and transfer area matrix were obtained based on the Markov module in the IDRISI 17.0 software.

(2) The suitability atlas for different LUCC types is obtained by inputting data information, such as elevation, slope, and the fixed ecological red line to constrain and limit the transformation of different LUCC types, taking into account factors, such as the actual topographical and geomorphological conditions of the watershed and the development of urban areas.

(3) Based on the measured LUCC 2010, the modified transfer probability and area matrices, and the suitability atlas for each LUCC type transfer, 5 × 5 CA filter (a rectangular space within 5 km × 5 km around a cell has a significant effect on the change in the state of the cell) was used for 5 cycles to simulate the LUCC 2015 for the HRB. The CA-Markov model simulates that land use in 2040–2060 will maintain the trend in 2010–2015 and finally get the LUCC 2040–2060 for the HRB.

Typically used in studies on the accuracy of LUCC change simulation and the evaluation of the accuracy of remote sensing image interpretation, the Kappa coefficient can check the consistency of the simulated image results with the observed image data as a whole. The Markov model can extrapolate time series while the CA model can forecast the spatiotemporal dynamic evolution of complex systems, comprehensive utilization of both models may extrapolate the spatial changes of landscape patterns scientifically and reasonably. In this paper, the Kappa coefficient is utilized to assess the precision of land pattern evolution predictions. The calculation formula is as follows [54,55].

(15) $$\mathrm{Kappa}=\frac{\left(P_{0}-P_{c}\right)}{\left(P_{p}-P_{c}\right)}$$

where *P*_0_ is the proportion of correct simulations, *P_c_* is the proportion of correct predictions in the case of random model, *P_p_* is the proportion of correct predictions in the ideal case. Kappa < 0.4 indicates a low degree of similarity between the two images, when 0.4 ≤ Kappa ≤ 0.75, the two images are generally similar, and when Kappa > 0.75, the two images have a significant consistency, indicating a good simulation effect.

Among them, the parameter *n*, according to the Budyko equation, is mostly related to subsurface circumstances such as land use. An attempt was made to establish the empirical relationship between land use type and model parameter *n* in the HRB, so as to reveal the quantitative relationship between land use and model parameters and to identify the influence of land use change on runoff. Because forestland, grassland, and farmland occupy more than 95% of the total area of the HRB, this study exclusively considers these three land use types for the empirical equation of the model parameter *n*.

(16) $$n_{t}=\beta_{1}x_{1}+\beta_{2}x_{2}+\beta_{3}x_{3}$$

where $n_{t}$ is the parameter in the Budyko equation at time *t*; $\beta_{i}$ and is the regression parameter of each land use type; $x_{i}$ is the percentage of land use types (*i = 1*,..., *m*). Based on *P*, *R* and *E*_0_, the Budyko parameters *n* (six 5-year periods) were inversed against the HRB during the period 1980–2014, and the $\beta_{i}$ was fitted by multiple linear regression.

## 3. Results

### 3.1. Historical Hydrometeorological Analysis and Attribution Analysis

#### 3.1.1. Assessment of Climatic and Hydrological Variables during 1974–2014

**Figure 3:**
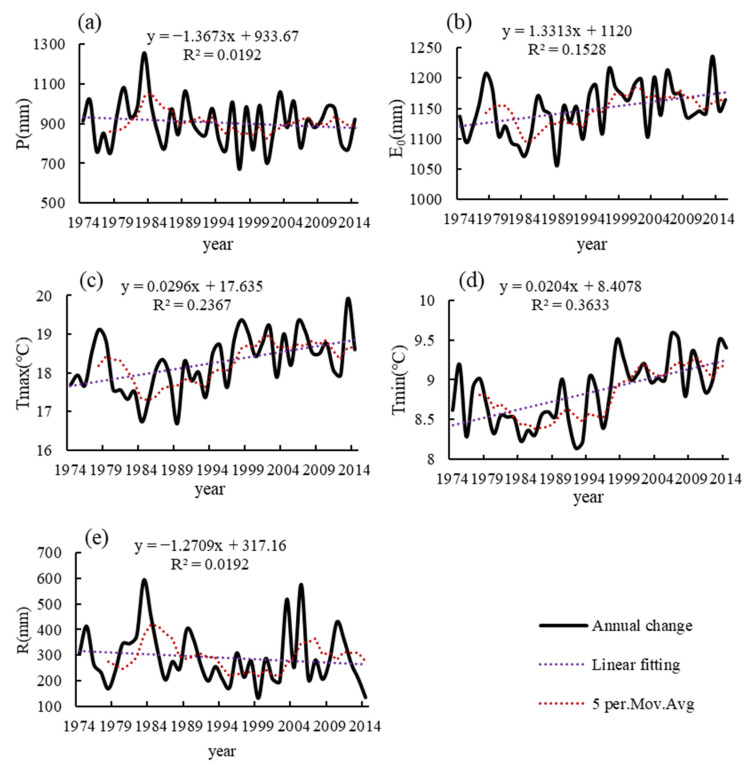
Change trends in climate and hydrology in the HRB 1974–2014: (**a**) average annual precipitation (*P*); (**b**) average annual potential evapotranspiration (*E*_0_); (**c**) average annual maximum temperature (*Tmax*); (**d**) average annual minimum temperature (*Tmin*); (**e**) average annual runoff depth.

To better understand the runoff processes during the historical period 1974–2014, linear regression and MK trend tests were used to analyze the trend of hydrometeorological series, as shown in Table 3. Figure 3 shows the linear fitting curves, annual mean lines, and 5-year sliding averages of hydrometeorological variables in the HRB during 1974–2014. From the linear fit curve analysis, it can be seen that temperature and potential evapotranspiration show a non-significant upward trend, which is generally consistent with the results of the MK trend test, increasing at the rates of 0.0296 °C/a, 0.0204 °C/a and 1.3313 mm/a, respectively. While *P* and *R* show a downward trend, which is generally consistent with the results of the MK trend test, decreasing at the rates of 1.3673 mm/a and 1.2709 mm/a. The maximum values of average annual *P* and *R* from 1974 to 2014 occurred in 1983, at 1255.8 mm and 593.6 mm, respectively. The fluctuations of *E*_0_ were roughly the same as those of temperature. From 1974 to 2014, the average annual *P* was 906.97 mm, the average annual *E*_0_ was 1061.47 mm, and the average annual *R* was 290.47 mm. The potential evapotranspiration is higher than the precipitation in the HRB during the historical period.

**Figure 4:**
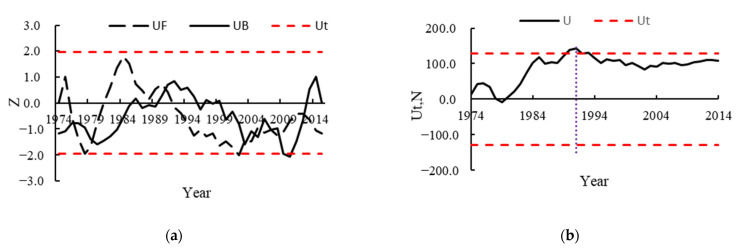
Runoff mutation analysis for 1974–2014 in the HRB: (**a**) M-K mutation test (**b**) Pettitt mutation test.

The MK test and Pettitt’s mutation test were used to determine the mutation years of the HRB runoff, as shown in Figure 4, to better attribute the HRB runoff. The intersection of the MK test UF and UB curves between the two critical levels α = 0.05 was first used to determine the year of mutation, and then, the Pettitt test was used to further verify the reasonableness and significance of the MK test for the year of mutation. The UF curve of the average runoff series of the HRB from 1974 to 2014 shows irregular fluctuations, with a decreasing trend from 1984 to 2002, though they are within the confidence interval of the significance level α = 0.05 (−1.96). This indicates a decreasing trend in the HRB runoff in these years, but the decrease is not significant. The UF and UB curves in the confidence interval intersected between two significance level lines in 1979, 1991, 2003, 2007, and 2008, preliminarily identifying the year of mutation, while the *U_t_*_,__*N*_ curve after Pettitt test identified the year of mutation of runoff in the HRB as 1991. Based on the abrupt change test, the study period 1974–2014 can be divided into two segments: the base period 1974–1991 and the change period 1992–2014, both of which provide a foundation for the subsequent attribution analysis.

#### 3.1.2. Analysis of Runoff Elastic Coefficient

According to the analysis in Section 3.1.1, it can be determined that the year of a sudden change of runoff in the HRB is 1991, which is consistent with the results of Peng Tao et al. [34]. Based on the results of the mutation analysis, the historical period was divided into the base period (1974–1991) and change period (1991–2014). Based on the average potential evapotranspiration, average runoff depth, and average precipitation of the two periods at Huangzhuang station, the corresponding Budyko parameter *n* of each period was calculated using Equation (3). Combining Equations (4)–(6) to calculate the elasticity coefficients $\varepsilon_{P}$, $\varepsilon_{E_{0}}$ and $\varepsilon_{n}$ corresponding to the two periods, we obtained the results as shown in Table 4.

The HRB has a subtropical monsoon climate that is both mild and humid. In comparison to previous studies, the *E*_0_ range is 800–1200 mm, the *P* range is 800–1900 mm, and *n* primarily ranges from 1.0 to 2.0 in China’s humid regions [56]. Comparing the two periods before and after the mutation, *P* decreased by 4.87% in the change period (1992–2014) compared to the base period (1974–1991), while potential evapotranspiration and *n* showed an increasing trend compared to the base period (1974–1991), increasing by 3.77% and 5.77%, respectively, or resulting in a 16.95% decrease in the runoff. The precipitation elastic coefficient $\varepsilon_{P}$, potential evapotranspiration elastic coefficient $\varepsilon_{E_{0}}$ and landscape elastic coefficient $\varepsilon_{n}$ in the change period (1992–2014) are 1.944, −0.944 and −1.026, respectively, indicating that when the *P* increases by 1%, the runoff will increase by 1.944%, the *E*_0_ will increase by 1%, it will lead to a 0.994% reduction in runoff, and when the Budyko parameter *n* increases by 1%, it will lead to a 1.026% reduction in runoff. It can be seen that the change in runoff at HRB is positively correlated with precipitation and negatively correlated with potential evapotranspiration and subsurface changes, which reflects the strong influence of climate on the change in the runoff. The absolute magnitude of the elasticity coefficient reflects the sensitivity of runoff to the various influencing factors. The effects of climate change and subsurface on catchment hydrology described above can also be explained by the Budyko curve. With the rise in drying index *E*_0_/*P*, the precipitation elasticity coefficient of runoff $\varepsilon_{P}$ will increase, while the potential evapotranspiration elasticity coefficient of runoff $\varepsilon_{E_{0}}$ will decrease. In comparison with the base period, $\left|\varepsilon_{P}\right|$ $\left|\varepsilon_{E_{0}}\right|$ and $\left|\varepsilon_{n}\right|$ increase during the change period, demonstrating an increasing susceptibility of runoff to changes in these three factors. Overall, runoff in the HRB is most susceptible to precipitation and least susceptible to changes in potential evapotranspiration.

#### 3.1.3. Runoff Attribution Analysis

Table 5 shows the contribution of each influencing factor to the change in runoff in the HRB. We can learn from Table 5 that the variations of runoff caused by precipitation, potential evapotranspiration and underlying surface are −29.34 mm, −10.66 mm, and −16.66 mm, respectively. Both climate change and human activities contribute to the decrease in the runoff, with precipitation changes accounting for 54.1%, subsurface changes accounting for 30.7%, and potential evapotranspiration accounting for 19.7% for the change in runoff. We can conclude that precipitation is the primary cause of decreased runoff in the HRB.

### 3.2. Climate Change Scenario Setting

#### 3.2.1. Evaluation of Statistical Downscaling and Bias Correction Results

**Figure 5:**
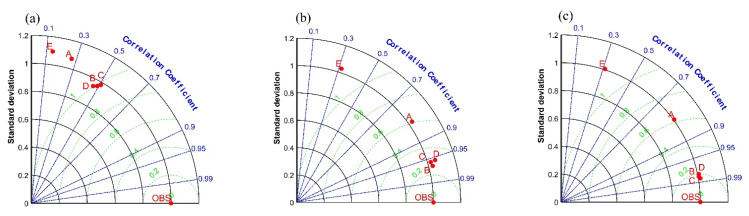
The 1961–2011 mean CMIP6 model-corrected Taylor diagram: (**a**) precipitation (*P*) (**b**) maximum temperature (*Tmax*) (**c**) minimum temperature (*Tmin*); OBS indicates measured values, A, B, C, D, E represent the five models CanESM5, MRI-ESM2-0, IPSL-CM6A-LR, NESM3, KACE-1-0-G, respectively.

Taylor diagrams of simulated *P*, *Tmax*, and *Tmin* versus observed ones were made to assess the ability of each CMIP6 model to affect measured data after bias correction. Figure 5 illustrates that after bias correction, the correlation coefficients for *P* are in the range of 0.1–0.6, with MRI-ESM2-0, IPSL-CM6A-LR correlation coefficients more than 0.4, mean squared deviation ratios at 0.98–1.01, and standard deviation ratios at 0.98–1.01. The correlation coefficients for *Tmax* after bias correction ranged from 0.3 to 0.99, with MRI-ESM2-0, IPSL-CM6A-LR and NESM3 having correlation coefficients greater than 0.95, ratios of mean squared deviations between 0.2 and 0.4, and ratios of standard deviations between 0.2 and 4. The correlation coefficients for the *Tmin* after bias correction ranged from 0.3 to 0.99, with MRI-ESM2-0, IPSL-CM6A-LR and NESM3 having correlation coefficients greater than 0.95, ratios of mean squared deviations between 0 and 0.2, and ratios of standard deviations between 0 and 0.2. By integrating the three indices, we can see that the order of CMIP6 models, after simulation and after bias correction, is *Tmax*, *Tmin*, and *P*, from strongest to worst. MRI-ESM2-0, IPSL-CM6A-LR, and NESM3 are the CMIP6 models offering better simulation ability after bias adjustment. Overall, it is found that the simulation of precipitation and temperature in the HRB by this correction method is better than the simulation of precipitation in the HRB. Nevertheless, the modeled average annual precipitation trends and multi-year averages are reasonably consistent with the observed values. This suggests that the correction method can be applied to future hydrological simulations of the basin and assess future runoff changes in the HRB.

**Figure 6:**
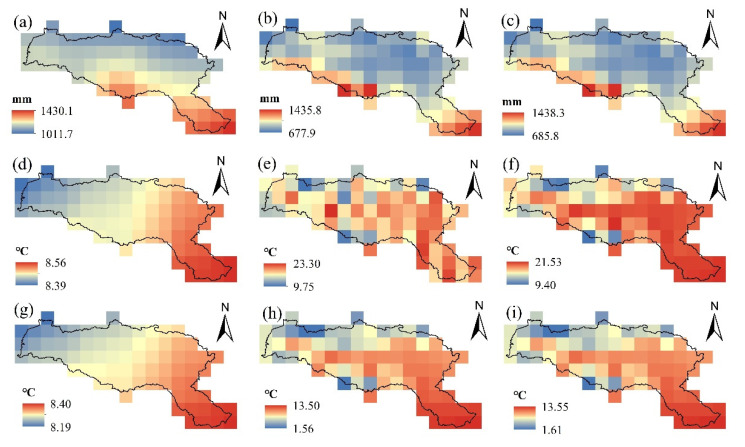
Spatial distribution between multi-model average simulated, corrected and observed values in 1961–2011: (**a**–**c**) pre-corrected multi-model average precipitation (*P*), corrected multi-model mean value, observed value; (**d**–**f**) pre-corrected multi-model average value of maximum temperature (*Tmax*), corrected multi-model average value, observed value; (**g**–**i**) pre-corrected multi-model average value of minimum temperature (*Tmin*), corrected multi-model average value, observed value.

The bias-corrected approach has a significant correcting effect on the regional distribution of *P*, *Tmax* and *Tmin*. The multi-model mean can reappear the pattern of decreasing annual mean precipitation and average annual temperature from southeast to northwest in the HRB by comparing bias-corrected simulated data with observed data from 1961 to 2011 (Figure 6). The correction for the multi-year mean values in the basin was good, with the mean *P* deviation reduced from 30.2% to 0.85%, the mean *Tmax* deviation reduced from 53.53% to 0.71%, and the mean *Tmin* deviation reduced from 5.68% to 0.57%. The effect of model bias correction on total annual precipitation and average annual temperature grid point bias correction was also compared. Before and after correction, the model *P* grid point correlation increased from 0.68 to 0.99, the *Tmax* grid point correlation increased from 0.62 to 0.83, and the *Tmin* grid point correlation increased from 0.75 to 0.99. For the simulation of the effect of precipitation and temperature extremes in the basin, it can be seen that the interval correction for *P* is from 1011.7–1430.1 mm to 677.9–1435.8 mm, the interval correction for *Tmax* is from 8.39–8.56 °C to 9.75–23.3 °C, and the interval correction for *Tmin* is from 8.19–8.4 °C to 1.56–13.5 °C, all of which are more in line with the actual values observed in the basin. The bias correction is effective with a good correlation in both space and time, and is anticipated to be applied in subsequent hydrological simulations of the basin.

#### 3.2.2. Analysis of Future Changes in Hydrological Variables

**Figure 7:**
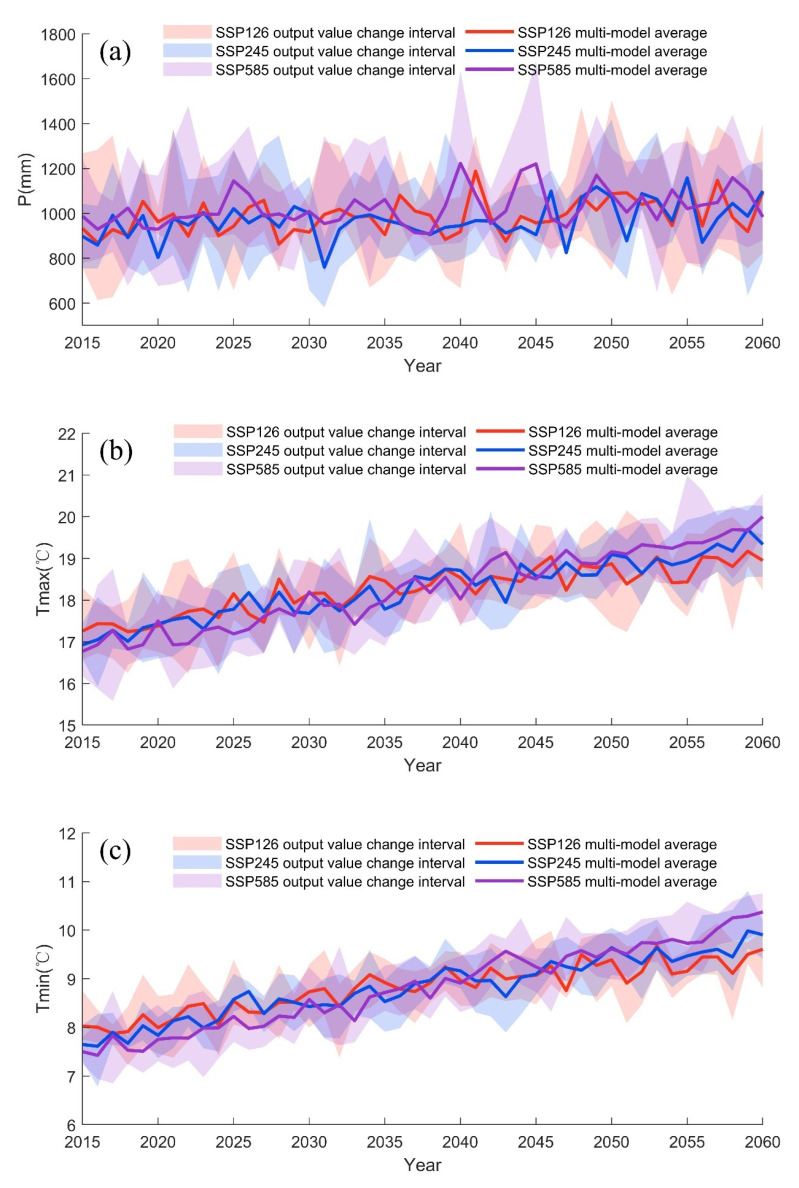
Hydrological variables predicted for 2015–2060 in the HRB: (**a**) average annual precipitation (*P*); (**b**) average annual maximum temperature (*Tmax*) (**c**) average annual minimum temperature (*Tmin*).

Figure 7 shows the average annual *P*, average annual *Tmax*, and average annual *Tmin* variations of the HRB in the future period under the SSP126, SSP245, and SSP585 scenarios. It can be seen from Figure 7 that the overall *P* is estimated to increase in the coming years, with the increase amplitude under SSP126 scenario > SSP585 scenario > SSP245 scenario, and the increase amplitude of *P* is estimated to enlarge with time. Temperatures are rising, with *Tmax* and *Tmin* increase amplitude under SSP585 scenario>SSP245 scenario >SSP126 scenario, and the increase amplitude is estimated to enlarge with time. The change rates of hydrological variables in the HRB relative to historical period (1974–2014) under different scenarios are shown in Table 6. Under the SSP126 scenario, *P* in the future period (2015–2040) increases by 22.38%, *Tmax* and *Tmin* decrease by 2.04% and 3.75% compared to the historical period (1974–2014). Under the SSP245 scenario, *P* in the future period (2015–2040) increases by 4.27%, *Tmax* and *Tmin* decrease by 2.25% and 4.89% compared to the historical period (1974–2014). In the SSP585 scenario, the *P* in the future period (2015–2040) increased by 10.64%, *Tmax* and *Tmin* decreased by 3.95% and 7.23% compared to the historical period (1974–2014). However, in the future period (2040–2060), its *P* increases by 22.86%, 10.57% and 16.96%, *Tmax* increases by 2.28%, 2.83% and 5%, and *Tmin* increases by 4.37%, 5.97% and 9.03% for SSP 126, SSP 245 and SSP 585 scenarios, respectively. Compared to the historical period (1974–2014), the increase in average *P* is more pronounced under the SSP126 scenario and the SSP245 scenario has the smallest increase. The SSP585 scenario has the greatest increase in multi-year average temperature, while the SSP126 scenario has the smallest increase.

### 3.3. Land Use Change Scenario Setting

**Figure 8:**
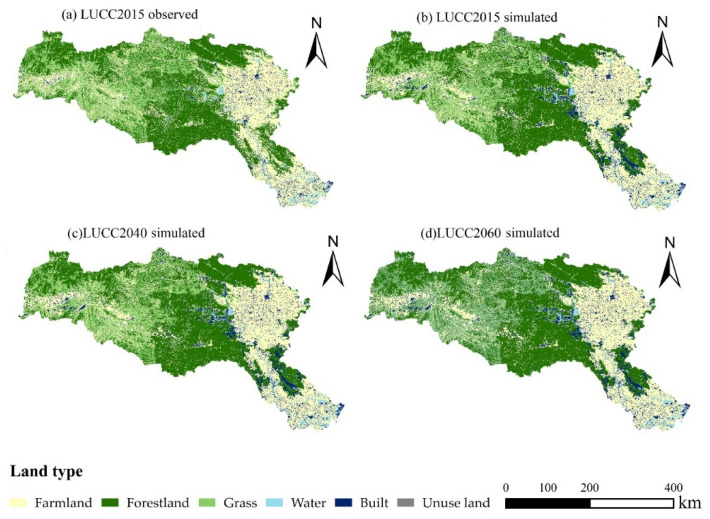
Land use in the HRB: (**a**) LUCC2015 observed; (**b**) LUCC2015 simulated; (**c**) LUCC2040 simulated; (**d**) LUCC2060 simulated.

Based on LUCC2010 data, the land use transition probability and adaptability atlas of LUCC2005-LUCC2010 were input into the CA-Markov model to predict LUCC2015, LUCC2040 and LUCC2060. The future land use simulation in the HRB is shown in Figure 8. Among them, the actual LUCC2015 (Figure 8a) and the simulated LUCC2015 (Figure 8b) were evaluated by the IDRISI 17.0 software, and the Kappa coefficient of the simulation result was 0.96, which confirmed that the model has a good prediction effect and the prediction results are credible. Based on this, further predictions were made for LUCC2040 (Figure 8c) and LUCC2060 (Figure 8d). According to the analysis of land use types in different periods of the HRB in Table 7, it can be seen that the changes of farmland, grassland and forest land between the measured LUCC2015 and simulated LUCC2015 land use types in the study area are not much different, which can accurately describe the land use situation in the study area and can be used for a follow-up analysis. At the same time, according to the analysis of the proportion of land use in LUCC2015, LUCC2040 and LUCC2060, it can be seen that from 2015 to 2040, the built areas in the study area increased from 3.12% to 7.34%, and the overall change from 2040 to 2060 was not significant. From 2015 to 2040, farmland and grassland will continue to decrease, from 34.95% to 32.78%, and from 19.48% to 17.54%, respectively. From 2015 to 2060, although there is a decreasing trend in forest land, the overall change is insignificant. It is worth noting that there is an increasing trend of unused land, indicating that there may be a trend of land degradation in the future.

According to the simulation results of LUCC2040 and LUCC2060 in Table 7 above, inputting the HRB model parameters, land use data into Equation (16), the multiple linear regression method was used to obtain an empirical formula as below, applicable to the study basin.

(17) $$n=-1.693x_{1}+15.602x_{2}-21.290x_{3}$$

Substituting future land use data into this equation, it can be determined that the *n* value is 1.866 for 2015–2040 and the *n* value is 1.871 for 2040–2060. They are then substituted into the Budyko framework to predict future runoff. After regression analysis, it was found that in the HRB, farmland and grassland had a negative effect on parameter *n*, while forestland had a positive impact.

### 3.4. Future Runoff Forecast

**Figure 9:**
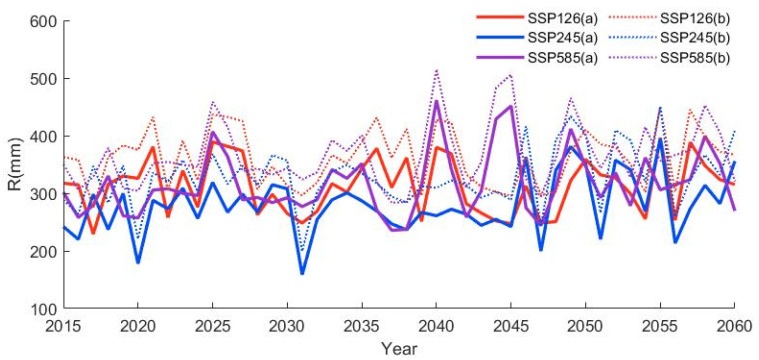
Annual runoff (*R*) predicted for 2015–2060 in the HRB; (**a**) 2015–2040: *n* = 1.866, 2040–2060: *n* = 1.871, (**b**) 2015–2040: *n* = 1.554, equivalent to the corresponding parameter *n* in 1992–2014.

**Figure 10:**
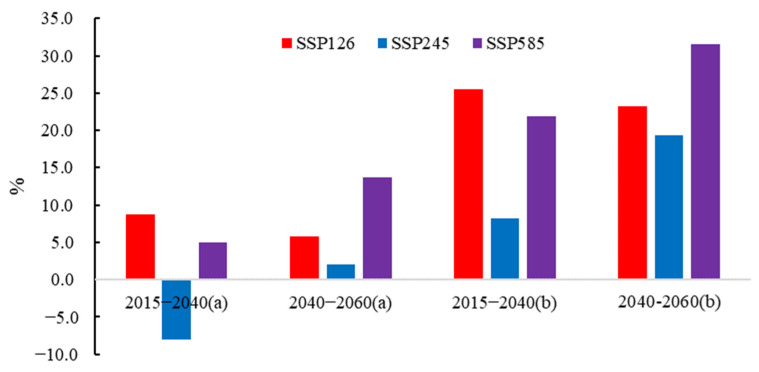
Rate of change of runoff (*R*) in different future periods (2015–2040, 2040–2060) against the historical period; (**a**) 2015–2040: *n* = 1.866, 2040–2060: *n*=1.871, (**b**) 2015–2060: *n* = 1.554, equivalent to the corresponding parameter *n* in 1992–2014.

This study uses an ensemble of climate models (multi-model averaging) from five CMIP6 GCMs combined with the Budyko water balance method to predict future runoff. We used Equation (17) to calculate the Budyko parameter *n* in the future period, see Section 3.3, which represents the land use maintenance LUCC2040 and LUCC2060 land use scenarios. The Budyko parameter *n* in 1992–2014 was calculated using Equations (1)–(3), shown in Table 3, representing the land use maintenance LUCC1992-2014 land use scenario. Based on Equation (3), the simulation forecast of the future runoff (*R*) of the HRB from 2015 to 2060 is carried out, as shown in Figure 9. According to Figure 9, it can be seen that the future *R* of the HRB will increase under the SSP126, SSP245 and SSP585 scenarios. The rate of change for future periods (2015–2040, 2040–2060) relative to historical periods (1974–2014) is shown in Figure 10.

To predict future runoff based on the Budyko water balance method, the most important thing is to determine the Budyko parameter *n*. This study estimates the Budyko parameter *n* based on land use scenarios. In Figure 10a, it is assumed that the land use maintains the LUCC2040 and LUCC2060 land use scenarios. Figure 10b assumes that the land use maintains the land use in the change period during 2015–2060. That is, the land use maintains LUCC1992-2014. Based on this, the effect of future runoff forecasting in the HRB was compared and analyzed. When the land use maintains the LUCC2040 and LUCC2060 land use scenarios, compared with the historical period (1974–2014), the runoff in the HRB increased by 8.77% under the SSP126 scenario from 2015 to 2040, and under the SSP245 scenario, the runoff in the HRB decreased 8.04%, under the SSP585 scenario, the runoff of the HRB increased by 4.97%, under the SSP126 scenario from 2040 to 2060, the runoff of the HRB increased by 5.79%, under the SSP245 scenario, the HRB runoff increased by 2.09%, under the SSP585 scenario. The runoff of the HRB increased by 13.66%.

When the land use is maintained under the LUCC1992-2014 land use scenario, compared with the historical period (1974–2014), the runoff in the HRB increased by 25.47% under the SSP126 scenario from 2015 to 2040, and under the SSP245 scenario, the HRB runoff increased by 25.47%. 8.27%, under the SSP585 scenario, the runoff of the HRB increased by 21.84%, under the SSP126 scenario from 2040 to 2060, the runoff of the HRB increased by 23.27%, under the SSP245 scenario, the HRB runoff increased by 19.35%, under the SSP585 scenario. The runoff of the HRB increased by 31.52%. When the land use is maintained under the LUCC1992-2014 land use scenario, compared with the historical period (1974–2014), the runoff in the HRB increased by 25.47% under the SSP126 scenario from 2015 to 2040, and under the SSP245 scenario, the HRB runoff increased by 25.47%. 8.27%, under the SSP585 scenario, the runoff of the HRB increased by 21.84%, under the SSP126 scenario from 2040 to 2060, the runoff of the HRB increased by 23.27%, under the SSP245 scenario, the HRB runoff increased by 19.35%, under the SSP585 scenario. The runoff of the HRB increased by 31.52%.

Overall, the future *R* of the HRB will show an increasing trend. Compared with the historical period, under the SSP126 scenario, the *R* increased significantly. Under the SSP585 scenario, the future *R* increase in the HRB is less. Under the SSP245 scenario, the future *R* variation in the HRB ranges from −8.04% to 19.35%. At the same time, by comparing the future *R* under the two ways, it can be found that the estimated future runoff of maintaining LUCC1974-2014 is generally higher than the *R* calculated by maintaining LUCC2040 and LUCC2060. Based on the assumptions in this paper, it is very likely to overestimate the future runoff in the HRB without considering the changes in the land use data of the underlying surface in the future.

## 4. Discussion

### 4.1. The Observed Impacts of Climate Change on Water Resources in the HRB

The current study results show that there is a decreasing trend for the annual runoff (*R*) and annual potential evapotranspiration (*E*_0_) in the HRB from 1974 to 2015, while there is an increasing trend for the annual temperature and potential evapotranspiration, overall consistent with the results of other studies [57]. According to the trend analysis in Section 3.1.1, the HRB experienced an extremely dry era following the 1990s, followed by a relatively dry spell in 2014.

Climate change and human activities lead to changes in key elements of the water cycle, such as precipitation, temperature, and substratum, in the future. The current study forecasts an increasing trend in overall precipitation and temperature in the future period. This is generally consistent with the results of other studies [58,59]. It has been shown that there is a consistent correlation between annual temperature and runoff, with a global temperature increase of 1 °C resulting in a 4% increase in runoff [60]. In this paper, an attribution analysis based on the Budyko equation for the historical period of the HRB can also further corroborate the influence of three factors on the future runoff of the HRB [61].

According to Zhai et al. [62], climate change is the most important risk factor for the hydrological risk of water supply in the South-North Water Transfer Central Line. According to Li et al. [63], climate change leads to a 15% reduction in runoff in the upper Han River. Meanwhile, climate change affects water quality and ecosystems. For example, increased temperature in climate change can lead to algal blooms in the Han River [64], while reduced flow can lead to deterioration of water quality in the middle and lower reaches of the Han River [65]. If this situation continues consistently, it will seriously affect the water transfer and transmission process in the HRB and have a serious impact on the South-North Water Transfer Project in China. This study provides some insights into the development of water resources and water quality management in the South-North Water Diversion by conducting an attribution analysis of the declining runoff in the HRB.

### 4.2. LUCC Change Impacts on Watershed Water Resources

Regarding the effect of LUCC on runoff variability, this is related to the parameter *n* in the Choudhury–Yang equation, where LUCC variability changes the vegetation retention, soil water content, and surface evapotranspiration involving hydrological factors [60]. Therefore, in this study, we assume that the change of parameter *n* is related to LUCC, etc. According to the actual LUCC in the HRB, which is mainly forestland, grassland and farmland, and the area of three land use types accounts for more than 95% of the total area of the HRB, so in this paper, we assume that the Budyko parameter *n* is related to three land use types in LUCC.

In a previous study, it was found that vegetation cover can weaken the water supply capacity of the South-North Water Transfer to some extent. For example, Zhang et al. [66] found that vegetation greening may exacerbate the degree of hydrological aridity. The increase in forest cover can reduce runoff in the HRB by as much as 0.19%, thus affecting river health to some extent [63]. In addition, the analysis of LUCC effects on water resources in the HRB may provide some suggestions for crop production in the HRB. Vegetation cover can, to some extent, weaken the water supply capacity of the South-North Water Transfer. The greening of vegetation may aggravate the severity of hydrological drought. For example, cotton crops in the HRB are highly dependent on irrigation water and are highly affected by the South-North Water Transfer Project [67].

According to the analysis of land use transfer changes in the HRB in Table 8, the number of transfers of the 3 types of land use, farmland, water and built, was the most obvious from 1980 to 2015. The net transfer out of farmland is 1876 km^2^, and the net transfer in water and built is 1092 km^2^ and 996 km^2^, respectively. The transfer of farmland to water is the highest transfer, 1064 km^2^, and built is mainly converted from farmland, 937 km^2^. There is a decreasing trend of forestland and grassland, which is not apparent.

Overall, land use changes will likely lead to significant changes in evapotranspiration in the HRB, which in turn will lead to a decrease in HRB runoff. In this study, there is a trend of decreasing grassland, forested and farmland in the future period, indicating a decrease in future evapotranspiration, further corroborating the increase in future runoff [60]. As future climate warming may lead to an increase in future evapotranspiration, it affects the availability of water resources. Therefore, this study predicts future runoff in the HRB can provide a basis for future water resources management and can also promote benefits such as soil and water conservation and ecological restoration.

### 4.3. Limitations of This Study

Of course, there are also some limitations in the experiment design. This study, for example, exclusively evaluates the effect of land use change on the subsurface parameter *n*, neglecting the impact of other hydrological variables. The currently available studies assume that the parameter *n* is related to climate change, vegetation, and many other factors, such as mean storm depth, precipitation seasonality, soil, vegetation cover [61,68], etc. In addition to this, there are interactions between various factors, such as the interaction between CA (farmland area as a percentage of total watershed area) and ASD (average storm depth). Many models (e.g., multiple stepwise regression, neural networks) [69] have been proposed to estimate the Budyko parameter *n* under specific conditions. In addition, during the land use simulation, all data were processed to a spatial resolution of 1 km, without considering the scale effect of land use. Further research is needed on how to maximize the mechanisms of natural and human influences on the geospatial system [70]. Therefore, in future studies, the impact of climate change and human activities on runoff should be input into the model to provide a more realistic picture of future runoff predictions in the HRB. The results expected to provide a basis for managing water resources in the changing environment of the HRB.

## 5. Conclusions

The impact of climate change and land use change on the reduction of runoff in the HRB was first analyzed, followed by an attribution analysis of the HRB based on the Budyko runoff elasticity coefficient method and a prediction for future runoff in the HRB based on the Budyko water balance method in combination with CMIP6 global climate model data. The following conclusions can be derived from the study’s findings:

(1) From 1974 to 2014, annual runoff and annual *P* in the HRB decreases non-significantly, with decline rates of 1.3673 mm/a and 1.2709 mm/a, respectively, whereas temperature and potential evapotranspiration increases non-significantly. Based on the mutation test, the year of mutation is confirmed to be 1991. According to the attribution analysis, precipitation is considered as the most critical factor leading to the drop in Han River runoff, with a contribution rate of 54.1%, followed by the lower bedding surface with a contribution rate of 30.7%.

(2) The overall simulation effect of temperature in the HRB after bias correction is better than *P*. The simulated annual average *P* trends and multi-year averages are reasonably consistent with the observed values, indicating a good spatial correlation. For the analysis of the bias-corrected future hydrological data, the overall *P* trend in the future period is increasing, with the increase amplitude under SSP126 scenario > SSP585 scenario > SSP245 scenario, and the precipitation increase amplitude also increases with time. Temperatures are estimated to rise, with *Tmax* and *Tmin* rises in the SSP545 scenario > SSP245 scenario > SSP126 scenario, and the temperature rise amplitude increases with time.

(3) For the future land use evaluation, there is a continuous trend of decreasing farmland and grassland in the future. Forestland has a decreasing trend, though the overall change is not significant. It is worth noting that there is an increasing trend of unused land, indicating that there may be a trend of land degradation in the future. The *n* value is determined to be 1.866 for 2015–2040 and 1.871 for 2041–2060.

(4) The future *R* of the HRB will show an increasing trend. The future runoff of the HRB shows an increasing trend, and the future runoff varies in different scenarios and periods. Under the land use scenarios of maintaining LUCC1992-2014 and LUCC2040 and LUCC2060, the *R* change rates in 2015–2040 are 8.27–25.47% and −8.04–19.35%, respectively, and the *R* in 2040–2060 are 2.09–13.66% and 19.35–31.52%. At the same time, by comparing the future *R* under the two scenarios, it can be found that the estimated future runoff of maintaining LUCC1992-2014 is generally higher than the *R* calculated by maintaining LUCC2040 and LUCC2060. Based on the assumptions in this paper, it is very likely to overestimate the future runoff in the HRB without considering the changes in the land use data of the underlying surface in the future.

## Figures and Tables

**Table 1:** Basic information on the five global climate models in CMIP6.

Model	Research Institutions	Country	Resolution (Lon × Lat)
CanESM5	Canadian Environment Agency (CCCma)	Canada	2.8125° × 2.8125°
MRI-ESM2-0	Meteorological Research Institute, Japan Meteorological Agency (MRI)	Japan	1.875° × 1.875°
IPSL-CM6A-LR	Pierre-Simon Laplace Institute (IPSL)	France	2.5° × 1.259°
NESM3	Nanjing University of Information Technology (NUIST)	China	1.875° × 1.875°
KACE-1-0-G	Institute of Meteorology, Korea Meteorological Administration (NIMS-KMA)	Korea	1.875° × 1.25°

**Table 2:** Hydrological variables and definitions.

Abbreviation	Definition	Units
*Tmax*	Maximum temperature	°C
*Tmin*	Minimum temperature	°C
*E*_0_	Potential evapotranspiration	mm
*P*	Precipitation	mm
*R*	Runoff depth	mm

**Table 3:** Results of hydrometeorological trend analysis.

Series	Linear Fitting	Z (MK)	Trend
*P*	−1.3673	−0.9174	down
*E*_0_	1.3313	1.2489	up
*Tmax*	0.0296	0.0303	up
*Tmin*	0.0204	0.0213	up
*R*	−1.2709	−1.5036	down

**Table 4:** Hydroclimatic characteristics of the HRB.

Data Period	Long-Term Mean Value					Elasticity of Runoff		
	Annual *P* (mm)	Annual *E*_0_ (mm)	Annual *R* (mm)	*E*_0_/*P*	*n*	$\varepsilon_{P}$	$\varepsilon_{E_{0}}$	$\varepsilon_{n}$
1974–1991	932.95	1039.05	319.99	1.11	1.469	1.882	−0.882	−0.900
1992–2014	887.49	1078.29	265.75	1.21	1.554	1.994	−0.994	−1.026
1974–2014	906.97	1061.47	288.98	1.17	1.515	1.942	−0.943	−0.969

**Table 5:** Analysis of runoff attribution in the HRB.

Period		Change from Base Period to Change Period				*P*/*E*_0_/*n* Induced Runoff Change (mm)			Contribution to Runoff Change (%)		
Base Period	Change Period	ΔR	ΔP	$\Delta E_{0}$	Δn	$\Delta R_{P}$	$\Delta R_{E_{0}}$	$\Delta R_{l}$	$\eta_{P}$	$\eta_{E_{0}}$	$\eta_{l}$
1974–1991	1992–2014	−54.24	−45.46	39.2	0.085	−29.34	−10.66	−16.66	54.1%	19.7%	30.7%

**Table 6:** Rates of change of hydrological variables in the HRB in future periods under different scenarios.

Period	2015–2040 (%)			2040–2060 (%)		
Variables	126	245	585	126	245	585
*P*	22.38	4.27	10.64	22.86	10.57	16.96
*Tmax*	−2.04	−2.25	−3.95	2.28	2.83	5.00
*Tmin*	−3.75	−4.89	−7.23	4.37	5.97	9.03

**Table 7:** Proportion of land use types (%) in the HRB in different periods (%).

Period	Farmland	Forestland	Grassland	Water	Built	Unuse Land
2015 observed	34.95	39.59	19.48	2.82	3.12	0.05
2015 simulated	32.93	39.46	17.53	2.75	7.20	0.13
2040 simulated	32.78	39.46	17.54	2.75	7.34	0.12
2060 simulated	32.76	39.49	17.55	2.75	7.30	0.14

**Table 8:** Land use area transfer matrix for the HRB 1980–2015 (km^2^).

Type of Land Use	Farmland	Forestland	Grassland	Water	Built	Unused Land	2015
Farmland	53,191	102	180	98	3	35	53,609
Forestland	136	60,397	169	16		1	60,719
Grassland	148	54	29,648	28		1	29,879
Water	1064	70	25	3057	5	100	4321
Built	937	165	32	29	3610	5	4778
Unuse land	9	1	2	1		64	77
1980	55,485	60,789	30,056	3229	3618	206	153,383

## Data Availability

Not applicable.
